# Corpus Callosum Microstructural Changes Correlate with Cognitive Dysfunction in Early Stages of Relapsing-Remitting Multiple Sclerosis: Axial and Radial Diffusivities Approach

**DOI:** 10.1155/2011/304875

**Published:** 2011-07-10

**Authors:** Carolina de Medeiros Rimkus, Thiago de Faria Junqueira, Katarina Paz Lyra, Marcel P. Jackowski, Melissa A. R. Machado, Eliane C. Miotto, Dagoberto Callegaro, Maria Concepción García Otaduy, Claudia da Costa Leite

**Affiliations:** ^1^Department of Radiology, Laboratory of Medical Investigation (LIM-44), School of Medicine, University of São Paulo (FM-USP), 05403-000 São Paulo, SP, Brazil; ^2^Department of Neurology, Clinical Hospital, School of Medicine, University of São Paulo (FM-USP), 05403-000 São Paulo, SP, Brazil; ^3^Department of Computational Sciences, Institute of Mathematics and Statistics, University of São Paulo (IME-USP), 05403-000 São Paulo, SP, Brazil; ^4^Department of Neurology, Division of Neuropsychology, Clinical Hospital, School of Medicine University of São Paulo (FM-USP), 05403-000 São Paulo, SP, Brazil

## Abstract

The corpus callosum is the largest fiber bundle in the central nervous system and it takes part in several cognitive pathways. It can be affected by multiple sclerosis (MS) early in the disease. DTI is capable of infering the microstructural organization of the white matter. The vectorial analysis of the DTI offers the more specific indices of axial diffusivity (AD) and radial diffusivity (RD), which have shown to be useful to discriminate myelin damage from axon loss, respectively. This study presents DTI results (mean diffusivity (MD), fractional anisotropy (FA), RD, and AD) of 23 relapsing-remitting MS patients and its correlation with cognitive performance. There were 47.8% of cognitive impaired patients (MS CI). We found signs of demyelination, reflected by increased RD, and incipient axon loss, reflected by AD increase, which was slightly higher in the MS CI. The cognitive changes correlated with the DTI parameters, suggesting that loss of complexity in CC connections can impair neural conduction. Thus, cognitive impairment can be related to callosal disconnection, and DTI can be a promising tool to evaluate those changes.

## 1. Introduction

Cognitive impairment is one of the major factors affecting the social functioning and quality of life of multiple sclerosis (MS) patients, being described in 45–65% of the clinical cases [1, 2]. The scales most commonly used for clinical classification of MS-related disability, such as the expanded disability status scale (EDSS) [3], are focused on locomotor dysfunction. Probably because of the multifocal characteristic of MS lesions, these traditional disability scales correlate poorly with cognitive disease-related changes in MS [4, 5]. In the last decade, it has been proposed to include the application of multimodality neuropsychological tests (NPT) in the assessment of clinical stage of MS, which resulted in a more comprehensive scale, the multiple sclerosis functional composite (MSFC) [6, 7]. 

Great efforts are being done in medical and biomedical studies aiming to design a map of cognitive functions [8–10]. However, given the complexity of cognitive pathways and cortical and subcortical connectivity, it is difficult to identify a single isolated domain for each cognitive skill. 

The corpus callosum (CC) is the major fiber bundle in the central nervous system (CNS) and plays a critical role in the interhemispheric communication, taking part in most of the cognitive pathways [5, 11, 12]. Its fibers connect the cortical and subcortical regions of the brain hemispheres interconnecting the auditory, sensory-motor, and memory information [13]. The CC is one of the sites most frequently affected by demyelinating lesions in MS, and the onset of those lesions happens very early in the disease [14, 15]. Additionally, CC atrophy is very common in MS [16, 17], and histopathologic studies have detected signs of axon loss and decreased fibers density in the CC of subjects with relapsing-remitting (RR) and secondary progressive (SP) MS [18]. There are also evidences that regional axonal loss in the CC is related to white matter lesions projected in the cerebral hemispheres [19, 20]. For all these reasons, it is conceivable that cognitive impairment in MS can be partially related to commissural disconnection [21]. 

Conventional MR imaging is useful to demonstrate MS macroscopic white matter (WM) lesions, but more recent research failed to prove direct relationship of clinical progression of the disability and brain atrophy with cumulative lesion volume [22]. In consideration with those findings, other factors such as Wallerian degeneration, axon dysfunction, and microscopic degeneration are gaining importance in the evaluation of the disease process, and several MRI techniques are being developed to access them [18, 23, 24]. 

Diffusion MRI techniques are based on the evaluation of random motion of water molecules and can provide insights about the tissue organization. DTI has evolved from those techniques, improving the evaluation of tissue microstructure and allowing the reconstruction of white matter tracts [25–27]. The main scalar parameters, obtained by DTI, fractional anisotropy (FA), and mean diffusivity (MD), are known to characterize alterations in myelin sheath and cell membrane integrity, but they are not specific for demyelination or axon loss. The coordinates of the tensor matrix can be diagonalized to extract the three Eigenvalues, *λ*_1_, *λ*_2_, and *λ*_3_, derived from the main eigenvectors of diffusion elements [26]. The combination of those eigenvalues defines two other and more specific parameters, the axial diffusivity (AD), parallel with the axon fibers, and the radial diffusivity (RD), perpendicular to axon fibers. The increases in those values are believed to represent axon loss and demyelination, respectively [25, 28].

The purpose of this study is to determine by DTI whether the microstructure of the CC is already altered in MS patients with short disease duration, and to establish if these DTI parameters are correlated with observed cognitive disability. 

## 2. Materials and Methods

### 2.1. Subjects

Twenty-three clinically defined MS patients (15 females) with relapsing-remitting course from an outpatient clinic were selected for the study. The mean age of the patient group was 31.95 ± 9.2 years (range 18–48 years), with a mean disease duration of 2.4 ± 1.4 years (range 0.4–4.74 years) and expanded disability status scale (EDSS) of 1.37 ± 1.2 (range 0–4.5).

Out of twenty-two, twelve patients received immunomodulatory therapy for at least six months (4 patients on glatiramer acetate, 3 on interferon beta-1a, and 5 on interferon beta-1b), without presenting significant side effects. All patients were out of clinical relapse and were steroid-free for at least three months prior to MRI examination.

A control group of twelve healthy volunteers (HC) (9 females) with age and educational level matched to our patients was also studied. The mean age of the HC was 27.75 ± 5.4 years (range 20–37 years), without significant difference with the MS patients (*P* = 0.12). There was no significant difference in the educational level between MS patients (14.23 ± 3.3 years of school education) and healthy subjects (14.87 ± 3.4 years of school education) (*P* = 0.87).

Subjects with significant depression measured by the Beck Depression Inventory (score 14 or higher) were excluded from the study. 

The study protocol was approved by the local ethical committee (CAPPESQ 0450/09). All subjects agreed to participate in the study by signing a written informed consent.

### 2.2. Magnetic Resonance and Diffusion Tensor Imaging

Brain MRI exams were performed in a 3.0 T magnetic resonance scanner (Intera Achieva, PHILIPS Healthcare, Best, The Netherlands) with an 8-channel head coil. Sixty to sixty-seven contiguous 2 mm axial slices were acquired covering the whole brain, using a diffusion-weighted spin-echo (SE) single shot echo planar imaging (EPI) sequence, with a *b-value* of 0 and 1000 s/mm², 32 diffusion encoding directions, field of view (FOV) = 256 × 256 mm, and a matrix size of 128 × 128.

**Figure 1:**
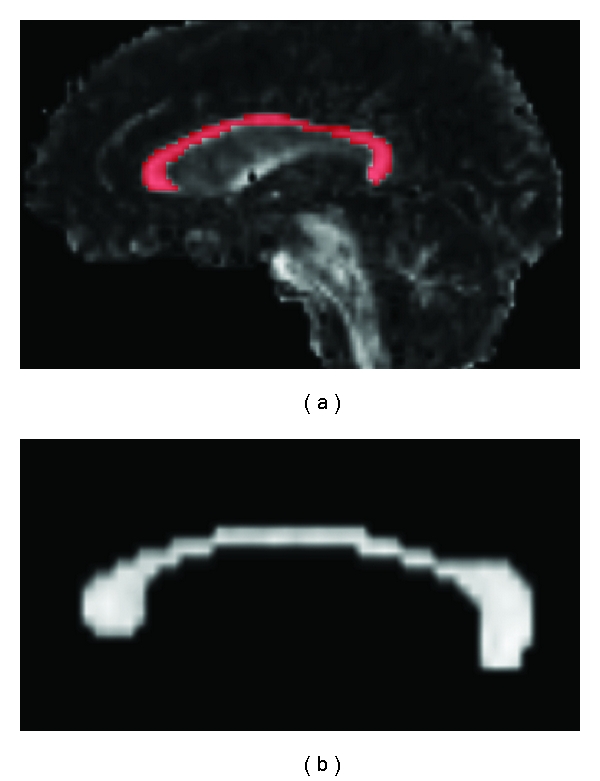
Sagital view of an individual FA map with the demarcation of the CC (a). In detail is the obtained CC mask (b).

Obtained DTI data were spatially coregistered with the scanner software and post processed with BioImage Suite 3.0 (http://www.bioimagesuite.org). Fractional anisotropy (FA), mean diffusivity (MD), and eigenvalue maps were generated for each subject. Two radiologists, C. M. Rimkus and K. P. Lyra, by consensus, manually delineated the medial section of the corpus callosum (CC) in the individual FA map defining the volume of interest (VOI) (Figure 1). The whole volume of the CC was included in the VOI mask, without lesion exclusion. Low anisotropy threshold of 0.2 was used to exclude voxels from adjacent structures or ventricles.

The axial diffusivity (AD) was represented by the main eigenvalue, parallel to the CC fibers, and the radial diffusivity (RD) was calculated from the mean of the two other eigenvalues, perpendicular to the CC fibers.

A fast spin echo (FSE) proton density-(PD-) weighted image (echo time (TE) = 20 ms; repetition time (TR) = 1775 ms; 28 contiguous 4.5 mm slices; matrix size = 320 × 320; FOV = 183 × 230 mm^2^) was used for lesion load analysis. An experienced neuroradiologist, C. M. Rimkus, performed the whole brain and CC manual segmentation of hyperintense lesions.

### 2.3. Neuropsychological Evaluation

The neuropsychological tests (NPTs) were administered by a skilled psychologist, M.A. R. Machado), in a single 2 h comprehensive evaluation where major domains of cognitive function were tested. Many of the tests used were from the Wechsler adult intelligence scale (WAIS-III) [29]. Briefly, the battery included tests of attention and information processing (Stroop, trail making test (TMT), symbol digit modalities test (SDMT) [30]), executive functions (Wisconsin card sorting test (WCST) [31], verbal fluency with phonemic association tests (FAS), and category association [32]), memory functions including short-term and working memory (Digit Span Forward and Backward and Letter-Number Sequencing from WAIS-III), long-term memory (Logical Memory, Hopkins verbal learning tests (HVLT) [33], Rey-Osterrieth complex figure (ROCF) delayed recall [34] and brief visual spatial memory test (BVMT) [35]), visuospatial functions (Block Design Test from WAIS III and ROCF copy), and verbal skills (Boston test and WAIS-III Vocabulary [29]). The intellectual function was also estimated by WAIS III-revised full-scale intelligence quotient (WAIS III-R FSIQ).

The individual score for each test was normalized based on the mean score and standard deviation of healthy adult population, obtaining the *z-score *[36]. The results below the 5th percentile were defined as abnormal.

Patients with three or more abnormal tests were classified as cognitive impaired.

No subject in the HC group was considered cognitive impaired, mild impairment was observed in four subjects for one task and in another one for two tasks.

The Beck depression inventory was also applied to exclude subjects with significant depression levels, which could interfere in the NPT.

### 2.4. Statistical Analysis

Descriptive statistics of demographic data such as age, sex, gender, and scholar level were analyzed to discriminate the distribution, frequency, and percentage of each of those variables in the sample and to characterize differences between the groups. Possible bias determined by the sample's demographic characteristics in the DTI parameters was tested by the Pearson's correlation test. This correlation test was also used to explore the correlation between DTI parameters and NPT and between them and MS patients' whole brain and CC lesion loads.

The MS patients were divided in cognitive impaired (MS CI) and cognitive preserved (MS CP). Mann-Whitney nonparametric *t*-test was used to evaluate significant differences in DTI parameters between MS patients and HC and between MS CI and MS CP. A *P* value <0.05 was considered significant.

The cognitive tasks were grouped by functional domain, and the relative frequency of abnormal tests in each domain in the MS patients' group was demonstrated. 

## 3. Results

### 3.1. Diffusion Tensor Imaging

In Table 1, DTI results obtained from the CC of MS patients, HC, MS CP, and MS CI groups and the respective lesion loads are shown. Compared to HC, MS patients presented in the CC significantly higher mean MD (*P* < 0.0001) and lower mean FA (*P* = 0.002). The RD (*P* < 0.0001) and AD (*P* = 0.009) were also significantly increased in MS patients. Both MS CP and MS CI presented significant differences in all DTI parameters compared to HC. The AD was slightly higher in MS CI compared to MS CP (*P* = 0.048).

**Figure 2:**
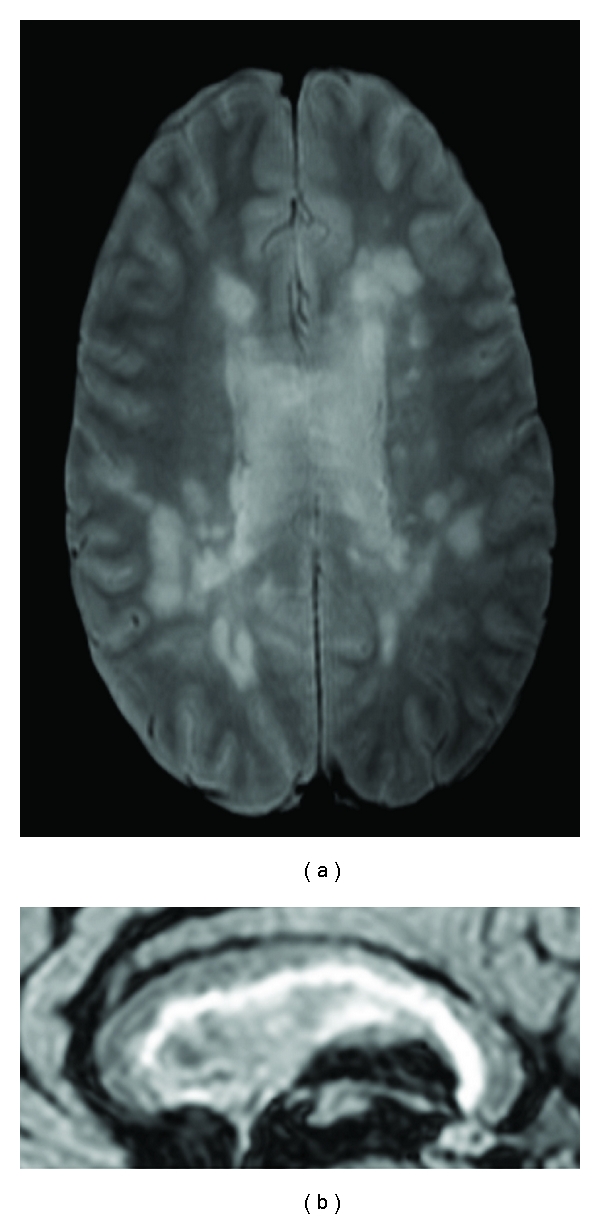
Female patient with high lesion load and no significant cognitive deficit. The PD axial image at the level of the body of the lateral ventricles and CC (a) shows lesions at the periventricular and subcortical white matter. The detail of mid-sagital FLAIR (b) shows the abundance of macroscopic lesion in the CC and callosal-septal interface.

**Figure 3:**
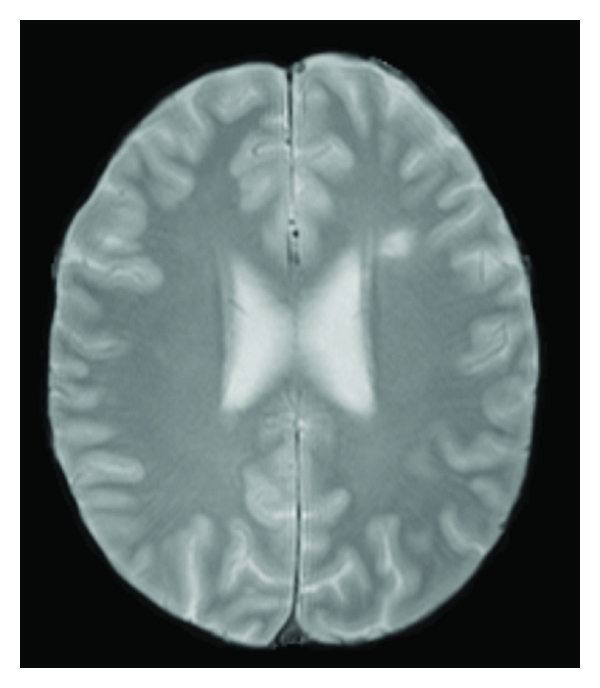
Female patient with low lesion load and significant cognitive deficit. The PD axial image illustrates the presence of few lesions in the supratentorial white matter, despite the cognitive deficit.

All patients, except one, presented lesions in the CC (range 0 to 35 lesions, mean 10.2 ± 7.7). There was no significant difference between MS CP and MS CI lesion loads (Figures 2 and 3). 

Pearson's test showed no correlation of DTI results and whole brain or CC lesion loads. There was also no correlation with disease duration, educational, or age level. A correlation of MD and RD with EDss (*P* = 0.03) was found (Table 2).

### 3.2. Neuropsychological Characteristics

Twelve patients (52.2%) were classified as MS CP and eleven (47.8%) as MS CI.  Table 3 summarizes the frequency of subjects with abnormal tests in each of the principal functional domains. In the most affected domains, the performance of the cognitive impaired patients was worse both qualitatively and quantitatively. MS CI had 1 to 3 tests below the 5th percentile of *z scores* in the sustained attention and information processing speed (IPS) domain (mean 1.7 ± 0.8) while MS CP had up to 1 test abnormal (mean 0.25 ± 0.45). In long-term memory tests, MS CI had up to 5 abnormal tests (mean 2.5 ± 1.5) while MS CP had up to 2 (mean 0.4 ± 0.7). In executive functions, MS CI had up to 2 abnormal tests (mean 0.5 ± 0.7) and in the MS CP, only one subject had 1 abnormal test (mean 0.1 ± 0.3).

Table 4 summarizes the correlation between the principal affected cognitive tasks and the DTI parameters.

The intellectual performances (WAISS III-R FSIQ) of the MS patients and HC were within the normal rates of the population.

There was no correlation between the NPT performances and the lesion loads.

## 4. Discussion

Cognitive decline can affect MS patients very early in the disease. In a group of MS patients with less than five years of disease, 47.8% were found to be cognitive impaired. The patients in this study presented deficits in sustained attention and IPS, long-term memory, and executive functions. The MS CI group showed also mild impairment in the short-term and working memory, visual-spatial, and construction tests.

The cognitive dysfunction was concomitant with DTI changes in the CC. MS group of patients showed decreased FA and increased MD compared to the matched HC, which can be interpreted as loss of complexity in the white matter tracts in the initial pathological process. The analysis of the principal eigenvalue (AD), parallel to axon fibers, and the average of the second and third eigenvalues (RD), perpendicular to fiber tracts, showed significant increase in RD and AD in MS patients.

It is believed that the increased RD reflects the demyelination degree while increased AD represents signs of axon loss [25, 28]. Although MS is classified as a myelin-related disease, several studies have reported loss of axons both in MS plaques as well as in the normal-appearing white matter (NAWM). Axon loss can be less prominent than demyelination, but its importance is recognized in the pathogenesis of symptoms [37, 38].

The observation of slightly increased AD in the MS CI patients reinforces the role of axon loss in the cognitive dysfunction. We neither have found significant differences in RD between MS CP and MS CI nor the DTI parameters have correlated with whole brain or CC lesion loads. Despite the fact that our relatively small sample can underestimate RD or lesion load differences, it is conceivable to hypothesize that other factors than demyelination itself or gliotic lesions can lead to cellular death and axon degeneration. Traditionally, the loss of axons in MS was associated with fiber transection due to inflammation and demyelination in the plaques, possibly conducting to Wallerian degeneration [38, 39], but more recent studies point that it might be an underlying diffuse axonopathy that contributes to the process of neurodegeneration [18, 20, 38].

The pattern of cognitive impairment is similar to the findings of previous studies [5, 40, 41]. Although there are some variations in the NPT results due to differences in the tests applied, the sample sizes, and patients' characteristics, the main abnormalities were found to be in sustained attention and IPS, executive functions, and memory domains. The functions more frequently related to CC microscopic lesion are in the attention and IPS and executive domains, suggesting anterior commissural damage with compromise of frontal and prefrontal pathways [20].

In this study, the stronger correlation was present between SDMT (attention and IPS) with FA (*P* = 0.02) and HVLT-delayed recall with RD (*P* = 0.03). The sustained attention and IPS domain presented other tests with a tendency to correlate with the DTI parameters: SDMT with MD and RD, TMT with AD, and Stroop test with FA and RD. The transverse characteristic of this study only provides a static picture of a dynamic process. It is not possible to predict the previous cognitive performance and the precise neuropsychological decline of the patients, and some of them might have greater functional reserve, diminishing the power of those correlations in this sample. Additionally, slight differences in the AD within the MS patients were present, which suggests different degrees of axon loss. These findings can reflect incipient heterogeneity of the sample, softening the direct correlations between DTI and NPT.

Neither the duration of the disease nor the age level of the patients correlated with the DTI parameters. Only subjects with short duration of the disease composed the sample; so, it was expected that this variable would not interfere in the image parameters. Previous studies showed that the peak of highest FA and lower MD in the CC happens between the ages of 21–44 years [42], and the main age-related DTI changes are described after 7th and 8th decades of life [43]. So, in our population with age range from 18 to 48 years, the DTI findings are more likely to be related with the MS disease than with the patients' ages.

However, MD and RD showed correlation with EDSS, suggesting possible relationship between callosal demyelination and sensory-motor dysfunction. The primary motor function takes part in the cortical spinal tract, but the inter-hemispheric communication of this pathway depends on midbody CC fibers [20, 38]. Hypothetically, the callosal disconnection can partially impair this neural conduction and influence the EDSS.

## 5. Conclusions

Cognitive impairment can be observed early in MS disease process, but it affects the MS population in different ways. The MS patients present signs of demyelination and loss of complexity of the CC, but patients with cognitive dysfunction might present earlier and more prominent axon loss, which could be related to incipient differences in the clinical and histological profile.

The cognitive decline is not directly associated to the macroscopic lesion load. Thus, advanced neuroimage studies are demanded to access microscopic abnormalities in the principal fiber tracts and functional pathways. The vectorial characteristic of the diffusion tensors makes it possible to explore signs of demyelination and axon loss, making of it a promising and noninvasive tool to evaluate MS pathological changes.

## Figures and Tables

**Table 1:** Results of DTI scalar maps (FA and MD) and vectorial diffusivity (AD and RD) in MS patients and healthy controls.

	MS Patients	MS CP	MS CI	HC
MD (10^−3^ mm^2^/s)	0.8611 ± 0.05	0.8492 ± 0.04	0.8741 ± 0.05	0.791 ± 0.04
FA	0.6879 ± 0.03	0.6899 ± 0.03	0.6858 ± 0.03	0.7206 ± 0.03
AD (10^−3^ mm^2^/s)	1.6788 ± 0.06	1.6591 ± 0.06	1.7003 ± 0.05	1.6102 ± 0.08
RD (10^−3^ mm^2^/s)	0.4487 ± 0.04	0.4451 ± 0.04	0.4528 ± 0.04	0.3818 ± 0.03
Whole brain lesion load	10.16 mL ± 9.8	9.54 mL ± 6.5	10.9 mL ± 12.8	—
CC lesion load	0.65 mL ± 0.7	0.77 mL ± 0.6	0.51 mL ± 0.7	—

**Table 2:** Pearson's correlation test evaluating possible correlations between DTI parameters and demographic characteristics of the sample.

	Age	Educational level	Duration of the disease	EDSS
MD	*r* = − 0.05	*r* = − 0.19	*r* = 0.37	*r* = 0.45*
FA	*r* = 0.22	*r* = 0.25	*r* = − 0.02	*r* = − 0.31
AD	*r* = 0.05	*r* = − 0.09	*r* = 0.29	*r* = 0.38
RD	*r* = − 0.09	*r* = − 0.25	*r* = 0.26	*r* = 0.45*

*Significant correlation (*P* = 0.03).

**Table 3:** Frequency of subjects with abnormal tests in the main cognitive domains in the MS patient subgroups.

	MS Patients	MS CP	MS CI
Short-term and working memory	8.7%	0.0%	18.2%
Long-term memory	60.9%	33.3%	90.9%
Executive functions	21.7%	8.3%	36.4%
Attention and information processing speed	60.9%	25.0%	100.0%
Verbal skills	0.0%	0.0%	0.0%
Visual spatial processing and construction	4.3%	0.0%	9.1%

**Table 4:** Pearson's correlation test between the main cognitive tasks and DTI parameters (considering *z scores* of all MS patients).

		MD	FA	AD	RD
HVLT-Immediate recall	*r*	0.09	0.25	0.12	−0.04
	*P*-value	0.7	0.2	0.6	0.9
HVLT-Delayed recall	*r*	0.33	−0.32	0.04	**0.45**
	*P*-value	0.1	0.1	0.8	0.03*
ROCF-Delayed recall	*r*	−0.10	0.15	−0.05	−0.014
	*P*-value	0.6	0.5	0.8	0.5
Logical memory I	*r*	−0.07	0.16	0.05	−0.21
	*P*-value	0.7	0.6	0.8	0.3
Logical memory II	*r*	−0.02	**0.41**	0.2	−0.21
	*P*-value	0.9	0.05**	0.3	0.3
Symbol Digit (SDMT)	*r*	−**0.37**	**0.46**	−0.17	−**0.37**
	*P*-value	0.07**	0.02*	0.4	0.07**
TMT	*r*	−0.31	−0.01	−**0.37**	−0.18
	*P*-value	0.1	0.9	0.08**	0.4
Stroop	*r*	−0.33	**0.36**	−0.12	−**0.39**
	*P*-value	0.1	0.08**	0.6	0.06**
FAS	*r*	−0.29	0.2	−0.17	−0.24
	*P*-value	0.2	0.3	0.4	0.3
Category association	*r*	−0.09	−0.04	−0.24	0.03
	*P*-value	0.7	0.9	0.3	0.9
Wisconsin	*r*	0.03	−0.15	−0.18	0.14
	*P*-value	0.9	0.5	0.3	0.5

*Significant correlation between cognitive test and DTI parameter.

**Cognitive tests that presented a tendency of correlation with the respective DTI parameter.
